# Commentary: Perceptions and attitudes toward clinical trial participation: a study on *Moringa oleifera* Lam. supplementation in adult HIV patients in Kano State, Nigeria

**DOI:** 10.3389/fphar.2026.1785490

**Published:** 2026-03-26

**Authors:** Benedict Kusi Ampofo, Marian Sapara Arthur

**Affiliations:** 1 Directorate of Internal Medicine, Komfo Anokye Teaching Hospital, Kumasi, Ghana; 2 School of Pharmacy and Pharmaceutical Sciences, University of Cape Coast, Cape Coast, Ghana

**Keywords:** clinical trials, patient and pubic involvement, sub-Saharan Africa (SSA), therapeutic misconception, trial participation

## Introduction

Clinical trials conducted in low- and middle-income countries frequently report challenges related to recruitment, retention, and protocol adherence (Tindana et al., 2007; Gul and Ali, 2010; Lang and Siribaddana, 2012). These challenges are commonly attributed to structural, logistical, and sociocultural factors that influence participation in research (Emanuel et al., 2004; Tindana et al., 2007). While descriptively useful, this framing underestimates the extent to which trials are shaped by participant expectations and practical constraints. These factors influence who enters trials, how participants engage with study procedures, and who remains under follow-up, with direct implications for trial validity and equity.

The study by Gambo *et al.*, examining perceptions and attitudes toward participation in a 6-month *Moringa oleifera Lam.* supplementation trial among adults living with HIV in Kano State, Nigeria, provides an opportunity to examine these issues more closely (Gambo et al., 2025). The intervention evaluated a bioactive plant product with antioxidant and immunomodulatory properties, positioning the trial within ethnopharmacology, complementary medicine, and infectious disease pharmacotherapy. The authors describe both motivations for participation and reasons for refusal, providing rare empirical insight into trial participation within routine HIV care. In this commentary, we examine how expectations of personal benefit and practical barriers may shape enrolment, influence trial conduct, and affect the interpretation and applicability of trial findings in sub-Saharan African settings.

## Discussion

### Self-benefit motivations and selection before randomisation

A striking feature of the study is the uniform prioritisation of self-benefit as a motivation for participation. All enrolled participants ranked “to help myself” and “to improve my health” as their most important reasons, with perfect mean scores and no observed variation (Gambo et al., 2025). Clinical trials are conducted because there is genuine uncertainty about benefit, and this uncertainty must also be clear to participants. When enrolment is driven by expectations of personal benefit, equipoise is effectively absent at the participant level. Individuals who believe the intervention will help them are more likely to enrol, while those who are uncertain, sceptical, or indifferent are less likely to take part. This creates systematic selection before randomisation: the randomised sample is shaped by participant expectations rather than drawn from the full eligible population.

Randomisation cannot correct this, because it balances only those who enter the trial and cannot reintroduce non-participants or restore uncertainty at the point of enrolment. As a result, the trial population is more likely to consist of participants predisposed to higher engagement, better adherence to study interventions, and greater retention, irrespective of allocation (Rothwell, 2005). While this does not threaten the internal validity of the study, it may influence external validity if participants who expect personal benefit from the intervention are more likely to enrol than those who do not. This difference between the trial population and the broader eligible population also has implications for how trial effects are interpreted. When participants enter a trial expecting personal benefit, adherence, symptom reporting, and retention may be influenced by expectancy and increased engagement rather than by the intervention alone. In such settings, observed effects reflect a combination of biological intervention effects and belief-driven behavioural responses, making attribution and interpretation more difficult (Heynemann et al., 2023).

### Structural barriers, selective enrolment, and equity

Non-participation in the study was driven primarily by time constraints and transport costs rather than by unwillingness to take part in research. Enrolment was therefore shaped by practical capacity to participate. Among individuals who were otherwise eligible, those with inflexible work schedules, caregiving responsibilities, or limited financial resources were less able to enrol, while those with greater availability and fewer constraints were more likely to do so. The authors note that transport stipends were available, yet financial constraints remained a barrier, suggesting that indirect costs (for example, lost income or caregiving arrangements) or incomplete understanding of available support may persist even when reimbursements are offered.

This pattern of selective enrolment has important implications for both trial interpretation and equity. The trial population is defined not only by eligibility criteria, but also by who is able to accommodate the demands of participation. As a result, individuals most affected by structural constraints, often those with greater socioeconomic vulnerability, are systematically under-represented, with consequences for the applicability and fairness of trial evidence (Bartlett et al., 2005; Rothwell, 2005). From an equity perspective, this raises concerns about whose experiences and outcomes are captured by trial evidence, and whose are excluded. Early engagement with patients, through a patient and public involvement (PPI) group, could have clarified what logistical support was needed, ensured that information on reimbursements was understood, and identified practical barriers such as time constraints, transport needs, caregiving responsibilities, or lost income. Such involvement can also shape the design of study materials and consent information, improving clarity around practical support and participation expectations. We recognise that structured PPI is not yet widely implemented in many LMIC research environments, but even informal engagement during study design can clarify logistical needs and improve the clarity and usability of participant information materials. These inputs could then inform pragmatic adaptations to study procedures, for example, by aligning visits with routine care or offering more flexible participation options. In this sense, PPI is not merely a recruitment strategy but a design mechanism for improving inclusivity and strengthening the relevance of trial evidence to real-world care.

The key participation challenges identified in the study and potential mitigation strategies are summarised in Table 1.

**TABLE 1:** Key participation challenges and possible mitigation strategies.

Challenge	Possible mitigation strategy
Expectation-driven enrolment	Clear communication of clinical uncertainty to potential participants during the consent process
Indirect participation costs (time, lost income, caregiving)	Early identification of barriers through consultation with patient and public involvement (PPI) groups and flexible scheduling where feasible
Logistical constraints (transport, visit timing)	Align study visits with routine clinical care where possible and provide transport reimbursement
Limited understanding of participation requirements	Improve participant information and consent materials through early PPI in study design
Under-representation of vulnerable groups	Early engagement with a well-constituted PPI group to identify structural barriers to study participation

### Implications for trial design and reporting in LMIC settings

The authors provide a careful and informative account of participant motivations and constraints within a completed clinical trial, offering data that are rarely reported from sub-Saharan African settings. Their findings show that participant expectations and practical barriers play a central role in shaping how trials are conducted and how their results should be understood. In settings where access to care is limited, and participation involves real time and financial costs, who enters a trial and why, has clear implications for both validity and equity. Early patient and public involvement, together with transparent reporting of who participated and who did not, can help address these issues and improve the relevance of trial findings to everyday clinical care (Crocker et al., 2018).

When recruitment and refusal data indicate that participation is being shaped by practical barriers, trials can adapt within protocol-defined limits to reduce avoidable exclusion, rather than discovering at trial end that key patient groups may have been systematically excluded. Treating recruitment and refusal data as signals for mid-course correction would strengthen recruitment, inclusivity and the interpretability of trial findings.

As plant-based and complementary agents continue to be evaluated in infectious disease settings, attention to participation dynamics will be essential for generating evidence that is both scientifically credible and relevant to the populations it is intended to serve.
